# Alternatives Assessment Frameworks: Research Needs for the Informed Substitution of Hazardous Chemicals

**DOI:** 10.1289/ehp.1409581

**Published:** 2015-09-04

**Authors:** Molly M. Jacobs, Timothy F. Malloy, Joel A. Tickner, Sally Edwards

**Affiliations:** 1 Lowell Center for Sustainable Production, University of Massachusetts Lowell, Lowell, Massachusetts, USA; 2 Sustainable Technology and Policy Program, School of Law, University of California Los Angeles, Los Angeles, California, USA; 3 Department of Community Health and Sustainability, University of Massachusetts Lowell, Lowell, Massachusetts, USA

## Abstract

**Background:**

Given increasing pressures for hazardous chemical replacement, there is growing interest in alternatives assessment to avoid substituting a toxic chemical with another of equal or greater concern. Alternatives assessment is a process for identifying, comparing, and selecting safer alternatives to chemicals of concern (including those used in materials, processes, or technologies) on the basis of their hazards, performance, and economic viability.

**Objectives:**

The purposes of this substantive review of alternatives assessment frameworks are to identify consistencies and differences in methods and to outline needs for research and collaboration to advance science policy practice.

**Methods:**

This review compares methods used in six core components of these frameworks: hazard assessment, exposure characterization, life-cycle impacts, technical feasibility evaluation, economic feasibility assessment, and decision making. Alternatives assessment frameworks published from 1990 to 2014 were included.

**Results:**

Twenty frameworks were reviewed. The frameworks were consistent in terms of general process steps, but some differences were identified in the end points addressed. Methodological gaps were identified in the exposure characterization, life-cycle assessment, and decision–analysis components. Methods for addressing data gaps remain an issue.

**Discussion:**

Greater consistency in methods and evaluation metrics is needed but with sufficient flexibility to allow the process to be adapted to different decision contexts.

**Conclusion:**

Although alternatives assessment is becoming an important science policy field, there is a need for increased cross-disciplinary collaboration to refine methodologies in support of the informed substitution and design of safer chemicals, materials, and products. Case studies can provide concrete lessons to improve alternatives assessment.

**Citation:**

Jacobs MM, Malloy TF, Tickner JA, Edwards S. 2016. Alternatives assessment frameworks: research needs for the informed substitution of hazardous chemicals. Environ Health Perspect 124:265–280; http://dx.doi.org/10.1289/ehp.1409581

## Introduction

Concerns about the impacts of toxic chemicals on the health of the public, workers, and ecosystems are receiving increasing scientific, business, and regulatory attention. From past scientific discoveries of harm, such as the neurotoxicity of lead or the carcinogenicity of vinyl chloride, to more recent concerns such as the range of potential adverse health outcomes associated with bisphenol A, today’s scientific journals and front-page media stories are documenting evidence of harm from chemicals that are widely used in commerce.

Although primary prevention by means of toxic chemical reduction and elimination is considered to be the most effective intervention to prevent morbidity and mortality associated with exposure, in the absence of a thoughtful evaluation of substitutes, “regrettable substitutions” can result [U.S. Occupational Health and Safety Administration (OSHA) 2015]. There are many recent examples of chemicals that were introduced as replacements for known toxic chemicals and were subsequently found to be toxic themselves. For example, in the late 1990s, 1-bromopropane (*N*-propyl bromide) was increasingly used as a drop-in replacement for known or suspected carcinogenic solvents such as methylene chloride and trichloroethylene [Centers for Disease Control and Prevention (CDC) 2008; Ichihara et al. 2012]. Within months of adopting 1-bromopropane as a drop-in replacement, case studies of severe neurotoxicity among workers quickly emerged (Reh et al. 2002). Not only is 1-bromopropane known to be highly neurotoxic, the National Toxicology Program (NTP) recently classified it as “anticipated to be a human carcinogen” (NTP 2014a). Because substitution of known toxic chemicals is an important public and environmental health prevention strategy, it is crucial to ensure that the selected alternatives will reduce human and environmental health risks. Adoption of a substitute, however, also depends upon its technical and economic feasibility.

Numerous governmental and private sector programs are driving a transition towards the substitution of hazardous chemicals with safer alternatives. Chemicals management regulations in the European Union (EU) and in states such as Washington, Maine, and California are requiring assessments of hazardous chemicals deemed “priority” or “very high concern” in order to evaluate the potential for safe and feasible substitution [European Parliament and Council 2007; Revised Code of Washington (Wash RCW) 2008; Maine Revised Statutes (Me Rev Stat) 2011; California Code of Regulations (CA Code of Reg) 2013]. Leading product manufacturers as well as major retailers have active chemical assessment and restriction policies and programs in place [Lavoie et al. 2010; National Research Council (NRC) 2014]. Central to many of these programs is the use of alternatives assessment.

Alternatives assessment is a process for identifying, comparing, and selecting safer alternatives to chemicals of concern (including those in materials, processes, or technologies) on the basis of their hazards, performance, and economic viability [Massachusetts Toxics Use Reduction Institute (MA TURI) 2013]. According to a recent National Academy of Science report, the goal of alternatives assessment is “ … to facilitate an informed consideration of the advantages and disadvantages of alternatives to a chemical of concern, resulting in the identification of safer alternatives” (NRC 2014).

Other terms are used for alternatives assessment, including chemicals alternatives assessment, alternatives analysis, or substitution assessment. A recent review conducted by the Organisation for Economic Co-operation and Development (OECD) noted that most definitions of alternatives assessment share a common focus on intrinsic hazard reduction and on taking action to replace chemicals of concern with safer alternatives (OECD 2013).

A number of alternatives assessment frameworks, guidance documents, and tools have been published by governments and nongovernmental organizations during the last decade, with some work dating back to the 1990s. In recent years, there have been efforts to develop detailed approaches, and there is a growing body of literature describing the practice and use of alternatives assessment in specific settings. Although alternatives assessments conducted in the business context are not routinely made publicly available and may not follow specific frameworks, dozens of alternatives assessments have been published, including those resulting from governmental programs or regulatory actions by government agencies. For example, numerous alternatives assessments have been conducted by industry as a result of “substance of very high concern” (SVHC) authorization regulatory requirements in the EU, and seven were conducted as alternatives assessment partnership projects of the U.S. Environmental Protection Agency’s (EPA’s) Design for Environment program (U.S. EPA 2015; Vainio 2015). Additionally, several state programs have published alternatives assessments on a wide range of toxic chemicals for specific applications [Interstate Chemicals Clearinghouse (IC2) 2015].

This substantive review provides a comprehensive overview of the literature on alternatives assessment frameworks. The purpose of this review is to identify consistencies and differences among published alternatives assessment frameworks as well as areas for future research and collaboration needed to advance this science policy practice. A recent National Academy of Sciences (NAS) report highlights the growing importance of alternatives assessment as a science policy discipline (NRC 2014). As when risk assessment was a new discipline, there is a need for scientific collaboration to identify where methods development is required to bring greater consistency in the field; at the same time, it is necessary to determine where flexibility and adaptability are appropriate given the particulars of the specific decision-making setting.

## Methods

This substantive review of alternatives assessment frameworks compares and contrasts how six standard components of an alternatives assessment are addressed. The six standard components as discerned by a preliminary review of the literature include *a*) hazard assessment, *b*) exposure characterization, *c*) life-cycle impacts consideration, *d*) technical feasibility evaluation, *e*) economic feasibility assessment, and *f*) decision making (i.e., how trade-offs among alternatives are evaluated and resolved).

Articles, reports, and web-based documents were searched using a variety of search tools, including EBSCO’s Discovery Service (http://www.ebscohost.com/discovery), which aggregates several literature databases or indexes, Medline, several Google search vehicles, and conversations with experts in the field. Search terms used included “alternatives analysis,” “alternatives assessment,” “chemical alternatives assessment,” “chemical alternatives analysis,” “chemical substitution,” “chemical substitution assessment,” and “technology options assessment.” The search was limited to literature published from January 1990 to December 2014. Literature eligible for the review included articles published in peer-reviewed journals or proceedings of professional societies, and reports and web-based resources produced by governmental and nongovernmental organizations and academic institutions. From the articles and reports that were initially identified, we selected a set of alternatives assessment frameworks for the literature review based on two criteria: *a*) The framework had to detail a multistep process for comparing chemical and design alternatives from options identification to assessment to implementation; and *b*) the framework had to include components considered central to an alternatives assessment—hazard assessment, economic feasibility, and technical feasibility. Papers that exclusively focused on an individual step in the alternatives assessment process (e.g., only chemical hazard assessment) were excluded. Papers and reports that only addressed policy aspects of alternatives assessment were also excluded, as were papers that simply described an alternatives assessment case study.

To enable a consistent review of the articles and reports that met the review inclusion criteria, a database was developed and used to extract and record methodological details for each of the six alternatives assessment components identified above. General information abstracted for all frameworks included *a*) year of publication, *b*) type of publication, *c*) authoring organization, and *d*) purpose of framework. Information abstracted for hazard assessment, economic and technical feasibility, exposure characterization, and life-cycle impact components included *a*) assessment end points, *b*) assessment methodology, *c*) data sources, and *d*) treatment of data gaps. For the decision analysis component, the information abstracted included *a*) decision function, *b*) decision approach used, *c*) decision tools used, and *d*) the role of weighting (each of these items is further defined in the results section). Assessment end points and measures were abstracted as described in a given alternatives assessment framework. The review of the information abstracted from a given alternatives assessment framework was limited by the extent to which the methodologies were described in the published framework.

## Results

*General characteristics.* The literature search identified a growing body of work of more than 200 articles and reports. Of these, 20 journal articles and reports (including online sources) outlining specific alternatives assessment frameworks met the inclusion criteria (multistep approach) and were included in this review (Table 1). Articles and reports that were identified in the search but not included in the review were in one of the following categories: commentaries about chemical substitution and alternatives assessment policy and practice or case examples; detailed reviews about specific tools used in alternatives assessment (e.g., hazard assessment tools); or documents that did not address the three essential components of an alternatives assessment: hazard assessment, economic feasibility, and technical feasibility. Regarding the last category, there were many studies that focused on only the assessment of hazards associated with alternatives or the life-cycle assessment of alternatives; these frameworks were excluded because they did not address essential components including cost and performance. Some organizations, such as the MA TURI and the University of California Los Angeles (UCLA) Sustainable Policy and Technology Program, have published multiple reports and/or articles on their alternatives assessment frameworks; in such cases, these frameworks were reviewed as a single entity (Eliason and Morose 2011; Malloy et al. 2011, 2013; MA TURI 2006).

**Table 1:** Alternative assessment frameworks reviewed and general characteristics (*n* = 20).

Framework name (reference)	Publication type		Publication source			Primary focus			Purpose			
	White paper/report/online source	Journal	Government agency	NGO	Academia	Chemicals management	Occupational health	Environmental protection	Regulatory	General guidance	Internal protocol	Research/case study
Goldschmidt 1993		✓			✓	✓				✓
U.S. EPA CTSA (Kincaid et al. 1996)	✓		✓			✓				✓	✓
Rosenberg et al. 2001		✓			✓		✓					✓
Lowell Center for Sustainable Production (Rossi et al. 2006)	✓				✓	✓				✓
MA TURI (Eliason and Morose 2011; MA TURI 2006)	✓	✓	✓			✓					✓	✓
P2OSH (Quinn et al. 2006)		✓			✓		✓					✓
Royal Society of Chemistry (RSC 2007)	✓			✓		✓				✓
TRGS 600 (BAuA AGS 2008)	✓		✓				✓		✓
UNEP Persistent Organic Pollutants Review Committee’s General Guidance on Alternatives (UNEP 2009)	✓		✓					✓	✓	✓	✓
U.S. EPA DFE Program (Lavoie et al. 2010; U.S. EPA 2011a)	✓	✓	✓			✓				✓	✓
BizNGO (Rossi et al. 2011)	✓			✓		✓				✓
German Guide on Sustainable Chemicals (Reihlen et al. 2011)	✓		✓			✓				✓
UCLA Sustainable Policy & Technology Program (Malloy et al. 2011, 2013)	✓	✓			✓	✓				✓		✓
REACH (ECHA 2011)	✓		✓			✓			✓	✓
U.S. EPA SNAP Program (U.S. EPA 2011b)	✓		✓					✓	✓
European Commission DGE (Gilbert et al. 2012)	✓		✓				✓		✓	✓
Ontario Toxics Use Reduction Program 2012	✓		✓			✓			✓	✓
OSHA 2013	✓		✓				✓			✓
Interstate Chemicals Clearinghouse (IC2 2013)	✓		✓			✓				✓
National Academy of Sciences (NAS) (NRC 2014)	✓		✓			✓				✓
NGO, nongovernmental organization.												

As shown in Table 1, the majority of frameworks reviewed were published as white papers or reports (*n* = 17). Thirteen of the papers were published by governmental agencies, such as the European Chemicals Agency, the MA TURI, and the U.S. EPA (Table 1). The remaining frameworks were published by nongovernmental organizations and academic organizations (*n* = 2 and *n* = 5, respectively). The primary purpose of the alternatives assessment frameworks reviewed was to provide general guidance (*n* = 15). However, as a result of legislative mandates for substitution of chemicals of high concern, six government agencies published alternatives assessment frameworks as part of regulatory directives, including the European Commission’s Directorate General for Employment, Social Affairs and Inclusion [referred to as the European Commission DGE (Gilbert et al. 2012) in text and tables] and the Committee on Hazardous Substances (AGS) of the German Federal Institute for Occupational Safety and Health [BAuA AGS) 2008] (see Table 1). Seven alternatives assessment frameworks were generated solely or partially for research purposes and/or for internal organizational decision making.

The alternatives assessment frameworks vary in terms of the methodological details, depth of description, and prescriptiveness. The majority of frameworks reviewed are not prescriptive protocols. Rather, they were developed as flexible guides for decision making. The methods outlined are often provided as examples, describing procedures that “could” be used, rather than “should” be used. A few frameworks in particular only provide guiding principles to be used across the various process components of an alternatives assessment (Goldschmidt 1993; Rossi et al. 2006, 2011). Although recently published frameworks contain more methodological detail than many of the early frameworks, they are nevertheless guides, not protocols (IC2 2013; NRC 2014).

Two frameworks offer options for each alternatives assessment process component within increasing levels of comprehensiveness. The framework developed by the IC2 offers multiple assessment levels within each process component (IC2 2013). The need for expertise, resource-intensive data sources, and data outputs increases as the the level increases. The European Commission DGE (Gilbert et al. 2012) framework offers options with increasing numbers of steps, degrees of complexity, and expertise needed for the most intensive option.

Although all of the frameworks reviewed focus on alternatives assessments for chemicals of concern, some are more focused on specific jurisdictions, sectors, or issues. Because of this focus, some frameworks are not as comprehensive as others with regard to including all process components. For example, a number of frameworks were developed as part of workplace health and safety initiatives, including research projects, programs, and regulatory directives. Among these initiatives are Quinn et al.’s Pollution Prevention–Occupational Safety and Health (P2OSH) framework, which was developed for use in worksite intervention programs; OSHA’s Transitioning to Safer Chemicals Toolkit, which provides web-based voluntary guidance on alternatives assessment for employers and workers; and the Technical Rules for Hazardous Substances 600 (TRGS 600) from the BAuA, which provides guidance to employers to meet their regulatory obligation regarding substitution processes for chemicals of concern (BAuA AGS 2008; Quinn et al. 2006; OSHA 2013). The strength of these alternatives assessment frameworks is their specific focus on the occupational setting. However, given that some of these frameworks do not address environmental impacts such as ecological toxicity, risk trade-offs could occur (see Table 2). Others, such as the United Nations Environment Program’s (UNEP’s) Persistent Organic Pollutants Review Committee for the Stockholm Convention on Persistent Organic Pollutants (POPs), focus specifically on related environmental impacts, such as ecological toxicity (Table 2), and other life-cycle considerations, such as impacts on greenhouse gas emissions or ozone depletion, rather than on occupational impacts (UNEP 2009).

**Table 2:** Hazard assessment end points (most frequently addressed, not comprehensive) (*n* = 20).

Framework name (reference)	Physicochemical							Human toxicity													Ecological toxicity				Other workplace hazards							
	C–CorrosivityEx–ExplosivityF/FP–Flammability/flash pointO–OxidizingR–ReactivityVP–Vapor pressureWS–Water solubility							AT–Acute mammalian toxicityC–CarcinogenicityD–DevelopmentalED–Endocrine disruption^*a*^E I/C–Eye irritation/corrosivityG–GenotoxicityM–MutagenicityN–NeurotoxicityOEL–Occupational exposure limits R–ReproductiveRSn–Respiratory sensitivitySI–Skin irritationSnS–Skin sensitivity													AqT–Aquatic toxicity B–BioaccumulationP–PersistenceW/T–Wildlife/ terrestrial ecotoxicity				Er–ErgonomicsExC–Excessive coldExH–Excessive heatN–Noise O–OdorR–RadiationS–Stress (demand/control)V–Vibration							
	C	Ex	F/FP	O	R	VP	WS	AT	C	D	ED	E I/C	G	M	N	OEL	R	RSn	SI	SnS	AqT	B	P	W/T	Er	ExC	ExH	N	O	R	S	V
Goldschmidt 1993								✓	✓			✓		✓		✓	✓	✓	✓	✓
U.S. EPA CSTA (Kincaid et al. 1996)	✓	✓	✓	✓	✓			✓	✓	✓		✓			✓	✓	✓	✓	✓	✓	✓	✓
Rosenberg et al. 2001									✓					✓	✓		✓	✓	✓						✓	✓	✓	✓	✓	✓	✓
Lowell Center for Sustainable Production (Rossi et al. 2006)
MA TURI (Eliason and Morose 2011; MA TURI 2006)	✓		✓		✓	✓	✓	✓	✓	✓		✓		✓		✓	✓		✓	✓	✓	✓	✓
P2OSH (Quinn et al. 2006)	✓		✓			✓		✓	✓			✓					✓	✓	✓	✓			✓		✓				✓
Royal Society of Chemistry (RSC 2007)									✓		✓						✓				✓	✓	✓	✓
TRGS 600 (BAuA AGS 2008)	✓	✓	✓		✓	✓		✓	✓			✓		✓	✓	✓	✓	✓	✓	✓	✓	✓	✓
UNEP Persistent Organic Pollutants Review Committee’s General Guidance on Alternatives (UNEP 2009)									✓	✓	✓			✓	✓		✓					✓	✓
U.S. EPA DFE Program (Lavoie et al. 2010; U.S. EPA 2011a)	✓	✓	✓	✓	✓			✓	✓	✓	✓	✓	✓	✓	✓		✓	✓	✓	✓	✓	✓	✓	✓
BizNGO (includes GreenScreen^®^) (Rossi et al. 2011)			✓		✓			✓	✓	✓	✓	✓	✓	✓	✓		✓	✓	✓	✓	✓	✓	✓
German Guide on Sustainable Chemicals (Reihlen et al. 2011)		✓	✓	✓		✓	✓		✓	✓	✓	✓		✓		✓	✓	✓	✓	✓	✓	✓	✓
UCLA Sustainable Policy & Technology Program (Malloy et al. 2011, 2013)		✓	✓	✓				✓	✓	✓	✓		✓				✓				✓			✓
REACH (ECHA 2011)^*b*^									✓					✓			✓
U.S. EPA SNAP Program (U.S. EPA 2011b)			✓			✓	✓					✓			✓	✓			✓		✓	^*f*^	^*f*^
European Commission DGE (Gilbert et al. 2012)	✓	✓	✓		✓			✓	✓	✓		✓		✓			✓	✓	✓	✓	✓	✓	✓		✓			✓				✓
Ontario Toxics Use Reduction Program 2012	✓	✓	✓		✓	✓		✓	✓	✓	✓			✓		✓	✓				✓	✓	✓	✓
OSHA 2013^*c*^	✓	✓	✓	✓	✓			✓	✓	✓	✓	✓	✓	✓	✓		✓	✓	✓	✓					✓			✓				✓
Interstate Chemicals Clearinghouse (IC2 2013)^*d*^			✓		✓			✓	✓	✓	✓	✓	✓	✓	✓		✓	✓	✓	✓	✓	✓	✓	✓
NAS (NRC 2014)^*e*^	✓		✓	✓	✓	✓	✓	✓	✓	✓	✓	✓	✓	✓	✓		✓	✓	✓	✓	✓	✓	✓	✓
These end points reflect those explicitly noted in the sources reviewed above either in lists or in the narrative.^***a***^The NAS and U.S. EPA DFE frameworks as well as frameworks using the GreenScreen® (CPA 2014), including IC2 and BizNGO include “Endocrine Activity” rather than “Endocrine Disruption” as an end point. ^***b***^The REACH framework references the use of physicochemical characteristics, although it does not specify which to evaluate. Beyond referencing CMRs (carcinogens, mutagens, and reproductive toxicants) there is not a list of specific health end points to consider in the REACH guidance document. ^***c***^The OSHA framework includes “use hazards” within the hazard assessment framework, including the physical form of the chemical as well as process/handling characteristics. ^***d***^The IC2 framework allows for different levels of assessment. End points noted reflect the most comprehensive level. Some occupational hazards (e.g., temperature) are captured in other assessment modules. ^***e***^The NAS framework includes physicochemical, health hazard, and ecotoxicity end points as different hazard assessment steps; persistence and bioaccumulation are included in the set of physicochemical end points, not ecotoxicity; wildlife toxicity in the NAS framework is broad and includes both terrestrial plants and animals. ^***f***^These end points captured under exposure characterization.																																

The following section reviews how each common process component—hazard assessment, exposure characterization, life-cycle impacts, technical feasibility, economic feasibility, and decision making—is addressed in the 20 different frameworks.

*Hazard assessment.* Hazard assessment is a primary component in all of the alternative assessment frameworks reviewed, but the level of detail and the methodology used to evaluate hazards varies. Broadly speaking, the hazard assessment component involves the assessment of chemical alternatives based on their inherent hazard properties. These hazard properties are then compared for the chemical of concern and the alternatives. The majority of the 20 frameworks outline specific hazard end points to be considered in an alternatives assessment. Table 2 outlines the most commonly addressed hazard assessment end points, which can be organized into four categories: *a*) physicochemical properties, *b*) human toxicity, *c*) environmental/ecological toxicity, and *d*) additional workplace hazards not captured in the aforementioned characteristics (such as ergonomic strain).

No single end point is consistently addressed across all of the reviewed frameworks. However, several end points are more frequently included than others (Table 2). For example, flammability is the most frequently included physicochemical characteristic (*n* = 14). Vapor pressure (*n* = 7), explosivity (*n* = 8), corrosivity (*n* = 9), and reactivity (*n* = 10) are included less frequently. Among human toxicity end points, carcinogenicity (*n* = 18), reproductive toxicity (*n* = 18), mutagenicity (*n* = 14), acute toxicity (*n* = 13), and skin irritation (*n* = 14) are most frequently included. Among ecotoxicity end points, aquatic toxicity (*n* = 13), persistence (*n* = 13), and bioaccumulation (*n* = 13) are most frequently included. The NAS framework considers persistence and bioaccumulation as physicochemical characteristics and goes beyond the majority of frameworks by also outlining the need to examine terrestrial ecotoxicity (i.e., toxicity to both plants and animals) (NRC 2014). Very few frameworks include additional workplace hazard characteristics; those that do include factors such as ergonomics (*n* = 4), noise (*n* = 3), and vibration (*n* = 2). The NAS framework is the only framework that considers the assessment of physicochemical hazards as a step prior to consideration of human health and ecotoxicity hazards, in order to focus the subsequent assessment steps (NRC 2014).

A variety of data sources were identified as the basis for information on hazard end points. Most frameworks offer examples of publicly available resources where information can be collected but do not suggest preferred sources or any data hierarchy wherein certain data types might be considered of higher value than others. The most highly referenced sources include Material Safety Data Sheets (MSDSs) or Safety Data Sheets (SDSs), authoritative scientific lists [such as the list of carcinogens from the International Agency for Research on Cancer (IARC)], regulatory or government priority chemical lists, publicly available substance and toxicity databases, and contact with manufacturers or the supply chain. Frameworks, including the BAuA’s TRGS 600, the German Federal Environment Agency’s Guide on Sustainable Chemicals, and the European Commission DGE framework, primarily use information from SDSs, notably the use of “H” (hazard) or “R” (risk) phrases associated with the Globally Harmonized System of Classification and Labeling of Chemicals (GHS) (BAuA AGS 2008; Gilbert et al. 2012; Reihlen et al. 2011). The NAS framework also elevates the use of GHS criteria and hazard descriptors wherever available (NRC 2014).

Very few frameworks offer methods for addressing incomplete hazard data for the hazard assessment element. The GreenScreen® hazard assessment method used in both the BizNGO and IC2 frameworks uses a “data gap” classification for end points for which there is insufficient information to assess the hazard [Clean Production Action (CPA) 2014]. This classification is considered in the overall grading (known as “benchmarks” in the GreenScreen® methodology), often resulting in a lower overall score (i.e., it is more cautious about hazard) (CPA 2014). When measured data are not available for some hazard end points, the U.S. EPA’s Design for the Environment (DFE) Program (Lavoie et al. 2010; U.S. EPA 2011a) and the European Chemical Agency’s (ECHA’s) Authorisation Guidance (ECHA 2011) under the Registration, Evaluation and Authorization of Chemicals (REACH) legislation use (quantitative) structure–activity relationships [(Q)SAR] to inform a hazard classification. The BAuA’s TRGS 600 also describes use of “the effect factor model,” which negatively weights substances for which toxicological data are missing (BAuA AGS 2008). The NAS framework describes the use of high-throughput data streams as a means to fill data gaps and eventually serve as primary data for end points of concern (NRC 2014).

Fifty percent (*n* = 10) of the hazard assessment approaches outlined in the frameworks use some type of comparative ranking or categorization scheme to help evaluate differences in the levels of severity among the hazard end points (e.g., high, moderate, or low). However, no dominant or consistent method is used. Metrics for each of the ranks are based on specific data sources ranging from continuous values [such as the lethal dose that kills 50% of the test sample (LD_50_)] to presence on an authoritative list to categorization based on a specific decision logic such as GHS classifications. Consideration of chemical potency (as well as the weight of the evidence, among other factors) is integral to the GHS hazard classifications (UN 2011). Thus, frameworks that have adopted the GHS classifications [such as the GreenScreen® method (used in the BizNGO and IC2 frameworks) as well as the framework of the U.S. EPA’s DFE Program] consider the potency of a chemical in eliciting a particular health end point in the hazard severity rankings (i.e., high, medium, low) (CPA 2014; Lavoie et al. 2010; U.S. EPA 2011a). Additionally, a number of hazard assessment tools, such as the GreenScreen® method, stratify hazard severity scores by route of exposure in order to provide additional insight into factors that influence a chemical’s ability to cause harm (CPA 2014).

Although there is some degree of consistency among frameworks regarding the metrics and associated criteria by which chemicals are ranked as higher or lower concern for each hazard end point, variation exists. For example, frameworks including those by BizNGO (using GreenScreen®), the German Federal Environment Agency, and the Ontario Toxics Use Reduction Program outline a three-point scale for carcinogenicity hazard ranking, whereas the U.S. EPA’s DFE Program framework outlines a four-point scale (Ontario Toxics Use Reduction Program 2012; Reihlen et al. 2011; Rossi et al. 2011; U.S. EPA 2011a). Data sources for the hazard rankings also vary. For example, the German Environment Agency framework outlines GHS risk phrases for the carcinogenicity rankings, whereas the BizNGO framework (using GreenScreen®, which is based on GHS methodology) includes over a dozen authoritative list sources for its carcinogenicity rankings (Reihlen et al. 2011; Rossi et al. 2011). It is unknown whether these differences in methods will result in differences in the outputs of the hazard assessment.

Regarding the other 10 frameworks that do not specifically include a hazard-ranking scheme, some do not specify any hazard characterization methodology (*n* = 4), some refer to established hazard assessment tools such as the Institute for Occupational Safety and Health of the German Social Accident Insurance’s (IFA’s) “Column Model,” GreenScreen®, or MA TURI’s “Pollution Prevention Options Analysis System” (P2OSys) (*n* = 4), and others reference using risk-based profiling methods (*n* = 2) (CPA 2014; IFA 2014; MA TURI 2014).

Several frameworks, including those from the U.S. EPA’s DFE Program, the Lowell Center for Sustainable Production, the Ontario Toxics Use Reduction Program, and BizNGO, are identified as “hazard-based” assessment processes, meaning these approaches make explicit the sufficiency of using primarily hazard data without the need for using specific data on exposure in selecting a safer alternative (Lavoie et al. 2010; Ontario Toxics Use Reduction Program 2012; Rossi et al. 2006, 2011). As Lavoie et al. (2010) noted, if an alternative imparts similar product and chemical use patterns as a chemical of concern, then exposure can generally be considered a constant; the risk can therefore be decreased from a reduction in chemical hazard. These frameworks and others, including the IC2 (2013) framework, order hazard assessment first in the overall assessment process to ensure that only those alternatives that demonstrate improved environmental and health attributes are further evaluated with regard to exposure, technical performance, cost, and so on. Frameworks from European organizations, including ECHA (2011), the Royal Society of Chemistry (RSC 2007), the European Commission DGE (Gilbert et al. 2012), and the BAuA’s TRGS 600 (BAuA AGS 2008), which were developed primarily in support of regulatory objectives, generally consider exposure in parallel with hazard in the substitution process and may include quantitative risk estimates. The NAS framework includes a comparative exposure step to elucidate how intrinsic exposure characteristics may modify the hazard profile of a substance (NRC 2014).

*Technical feasibility assessment.* Two categories of technical feasibility are characterized in the frameworks reviewed: *a*) technical feasibility, and *b*) issues associated with legal, labor, and/or supply chain feasibility. Within technical feasibility, two specific aspects are consistently present: chemical functional use, and performance or feasibility. Functional use (sometimes referred to as functional requirement or functionality) is included in all of the frameworks. Functional use refers to the purpose that a chemical performs or the properties that it imparts in a specific formulation, material, or product. For example, if the purpose of the chemical of concern is to provide solvency in a cleaning product or flame retardancy in a foam product, the alternative must achieve that same function. A few frameworks, including IC2 (2013), European Commission DGE (Gilbert et al. 2012), and OSHA (2013), include the concept of “necessity” in the evaluation of functional use requirements: if the chemical of concern does not provide a necessary purpose in the formulation, material, or product, or if specific performance is not necessary, then it may be eliminated, and performing an alternatives assessment may not be necessary. Although functional use/requirement is a prominent consideration, it is most often addressed early in the technical feasibility assessment process to reduce the number of candidate alternatives that achieve the same function as the chemical of concern to subsequently include in the full alternatives assessment. In addition to functional use, specific performance/quality characteristics of alternatives are addressed in 80% (*n* = 16) of the frameworks. These performance considerations include measures such as quality, reliability, durability, and usability. Other technical feasibility characteristics addressed in multiple frameworks include feasibility (including production and process changes) (*n* = 8) and consumer requirements (*n* = 8). Regarding other feasibility characteristics, supply chain availability (*n* = 4) and conformance with regulations/legal requirements (*n* = 8) are commonly referenced (Table 3).

**Table 3:** Technical feasibility assessment characteristics (most frequently addressed, not comprehensive) (*n* = 20).

Framework name (reference)	Technical feasibility						Legal/labor/supply chain feasibility		
	AS–Authoritative source (identified alternatives as feasible for application)CR–Consumer requirementsF–Feasibility FR–Functional requirements MR–Maintenance requirements P/Q–Performance/quality (includes measures such as reliability, longevity, durability)						Reg–Conformity with regulations/requirementsSC–Supply chain availabilityW–Worker perception/acceptance		
	AS	CR	F	FR	MR	P/Q	Reg	SC	W
Goldschmidt 1993				✓
U.S. EPA CSTA (Kincaid et al. 1996)		✓		✓		✓	✓
Rosenberg et al. 2001				✓	✓		✓		✓
Lowell Center for Sustainable Production (Rossi et al. 2006)				✓		✓		✓
MA TURI (Eliason and Morose 2011; MA TURI 2006)				✓		✓	✓
P2OSH (Quinn et al. 2006)			✓	✓		✓			✓
Royal Society of Chemistry (RSC 2007)				✓		✓
TRGS 600 (BAuA AGS 2008)		✓	✓	✓		✓	✓	✓
UNEP Persistent Organic Pollutants Review Committee’s General Guidance on Alternatives (UNEP 2009)				✓	✓
U.S. EPA DFE Program (Lavoie et al. 2010; U.S. EPA 2011a)				✓		✓
BizNGO (Rossi et al. 2011)				✓		✓
German Guide on Sustainable Chemicals (Reihlen et al. 2011)		✓	✓	✓		✓
UCLA Sustainable Policy & Technology Program (Malloy et al. 2011, 2013)				✓	✓	✓
REACH (ECHA 2011)		✓	✓	✓		✓	✓
U.S. EPA SNAP Program (U.S. EPA 2011b)			✓	✓
European Commission DGE (Gilbert et al. 2012)		✓	✓	✓		✓	✓	✓	✓
Ontario Toxics Use Reduction Program 2012		✓	✓	✓	✓	✓	✓
OSHA 2013		✓		✓	✓	✓	✓
Interstate Chemicals Clearinghouse (IC2 2013)^*a*^	✓	✓	✓	✓	✓	✓		✓
NAS (NRC 2014)				✓		✓
These end points reflect those explicitly noted in the sources reviewed above.^***a***^The IC2 framework allows for different levels of assessment. End points noted reflect all levels.									

Several frameworks, including IC2 (2013) and BizNGO (Rossi et al. 2011), note that availability of an alternative in the marketplace for similar applications may be sufficient to satisfy performance considerations. Three frameworks specifically include worker perceptions of the technical changes as specific attributes associated with the technical assessment process (Table 3).

The majority of the frameworks lack specificity regarding the methods or suggested data sources to address issues of technical feasibility. This lack of specificity is understandable given the varied context of performance considerations in evaluating alternatives. Most frameworks simply outline specific performance criteria and in some cases use a line of questioning to more explicitly detail the performance/technical needs and issues to be addressed [European Commission DGE (Gilbert et al. 2012); Rossi et al. 2006, 2011]. Among the frameworks that provide greater methodological detail, information sources for performance measures include conversations with stakeholders in the supply chain, published literature sources (including trade journals and scientific studies), and actual pilot testing (ECHA 2011; IC2 2013; Ontario Toxics Use Reduction Program 2012). Methods used to evaluate performance across alternatives primarily include the use of performance scales that vary from qualitative summaries (i.e., worse, same, better) and/or continuous measures from testing outputs compared with a range of tolerances as well as comparison with consensus standards and methods such as those published by ASTM International (http://www.astm.org/Standard/standards-and-publications.html), the International Organization for Standardization (ISO) (http://www.iso.org/iso/home/standards.htm), and others.

*Economic assessment.* Although all of the reviewed frameworks identify the need for an economic assessment of alternatives, not all include specific cost measures or methods. Two frameworks do not provide methodological details for the assessment, although each notes the importance of assessing costs (Goldschmidt 1993; Reihlen et al. 2011). Among the frameworks that did provide such detail, there are five general categories of economic measures, including commercial availability, direct costs, internal costs, external costs, and long-term costs (including assessments that capture economies of scale and value assessments associated with product innovation).

As described below, the majority of the frameworks include holistic cost assessments that encompass a range of direct and tangible indirect production costs, rather than simply comparing the alternatives with the chemical of concern in terms of product price. In general, the methods focus on the economic impact to a given firm because most of these frameworks were developed as guidance documents for the business/industry community. However, some frameworks include a broader perspective, such as the UCLA framework that also addresses the economic impact to consumers, and the UN POPs Committee framework that includes a more industry-wide economic impact perspective (Malloy et al. 2011, 2013; UNEP 2009). The NAS framework also acknowledges that in some situations, organizations conducting the alternatives assessment will not always be the same entity that executes the substitution; thus, financial information for a thorough economic assessment may not be available (NRC 2014).

As Table 4 shows, 45% of the reviewed frameworks (*n* = 9) include commercial availability considerations, and 30% of the frameworks (*n* = 6) also include sufficient quantity/supply available to meet demand. Regarding direct costs, the majority include manufacturing costs (*n* = 17), which includes costs associated with capital/equipment costs and chemical/material costs (including additional processing chemicals if needed). Other direct cost attributes include maintenance/storage (*n* = 12), end of life/disposal (*n* = 13), energy (*n* = 8), and employment and labor productivity (*n* = 11). Among the most frequently included nondirect manufacturing costs (indirect costs) are expenses associated with regulatory compliance, including industrial hygiene engineering controls and equipment, emissions controls (*n* = 11), and liability costs (*n* = 7), such as costs associated with spills, fires, explosions, worker compensation, and so forth. External costs or potential benefits noted in a handful of frameworks include economic impacts associated with factors such as product labeling, environmental impact costs, human health, or other life-cycle cost impacts such as costs associated with resource extraction. Eleven of the frameworks describe the need to include long-term financial indicators (e.g., net present value, internal rate of return, profitability index) to capture evolving, rather than static, pricing associated with factors such as economies of scale and the future value of product innovations.

**Table 4:** Economic assessment attributes (most frequently addressed, not comprehensive) (*n* = 20).

Framework name (reference)	Commercial availability		Direct costs							Indirect costs					External costs/benefits						Other
	CA–Commercial availabilityQ–Sufficient quantity availability		E–Energy costsEoL–End of life costsLP/E–Labor productivity/employment M–Manufacturing costs (chemical costs/equipment costs/additional processing chemical costs, etc.)M/S–Maintenance and storage costsT–Transition costs (including R&D)Tsp–Transportation costs							I–Insurance costs L–Liabilities (e.g., accidents, work days lost, cleanup)LT–Labor trainingRC–Regulatory complianceT/F–Taxes/fees					Env–Environmental impact costsHH–Human health impact costsOLC–Other life-cycle costs (e.g., extraction)PL–Product labelingPP–Public perceptionWM–Worker morale						LT-E–Long-term economic costs (economies of scale and product innovation worth)
	CA	Q	E	EoL	LP/E	M	M/S	T	Tsp	I	L	LT	RC	T/F	Env	HH	OLC	PL	PP	WM	LT-E
Goldschmidt 1993^*a*^
U.S. EPA CSTA (Kincaid et al. 1996)	✓	✓	✓	✓	✓	✓	✓	✓		✓	✓	✓	✓	✓	✓	✓		✓	✓		✓
Rosenberg et al. 2001					✓	✓						✓								✓
Lowell Center for Sustainable Production (Rossi et al. 2006)	✓			✓	✓	✓	✓				✓		✓		✓	✓					✓
MA TURI (Eliason and Morose 2011; MA TURI 2006)	✓	✓		✓		✓	✓						✓				✓				✓
P2OSH (Quinn et al. 2006)	✓			✓	✓	✓					✓	✓	✓
Royal Society of Chemistry (RSC 2007)	✓					✓															✓
TRGS 600 (BAuA AGS 2008)			✓	✓		✓	✓	✓	✓	✓		✓	✓					✓	✓	✓
UNEP Persistent Organic Pollutants Review Committee’s General Guidance on Alternatives (UNEP 2009)	✓	✓		✓	✓	✓	✓				✓		✓		✓	✓					✓
U.S. EPA DFE Program (Lavoie et al. 2010; U.S. EPA 2011a)^*a*^	✓
BizNGO (Rossi et al. 2011)				✓		✓	✓														✓
German Guide on Sustainable Chemicals (Reihlen et al. 2011)^*a*^
UCLA Sustainable Policy & Technology Program (Malloy et al. 2011, 2013)						✓															✓
REACH (ECHA 2011)^*b*^			✓	✓		✓	✓	✓			✓	✓	✓								✓
U.S. EPA SNAP Program (U.S. EPA 2011b)	✓	✓	✓		✓	✓	✓
European Commission DGE (Gilbert et al. 2012)		✓	✓	✓	✓	✓	✓		✓	✓		✓	✓			✓			✓	✓
Ontario Toxics Use Reduction Program 2012			✓	✓	✓	✓	✓	✓		✓	✓	✓	✓	✓					✓		✓
OSHA 2013				✓	✓	✓	✓	✓		✓	✓		✓	✓		✓			✓	✓
Interstate Chemicals Clearinghouse (IC2 2013)^*c*^	✓	✓	✓	✓	✓	✓	✓		✓				✓		✓	✓	✓	✓	✓	✓	✓
NAS (NRC 2014)			✓	✓	✓	✓		✓								✓					✓
These end points reflect those explicitly noted in the sources reviewed above. ^***a***^Cost assessment addressed in framework, yet no specific end points noted. ^***b***^The REACH framework states, “data may also be collected on … indirect benefits,” (p. 76) yet also states “impacts such as unemployment and health benefits are not considered part of the economic feasibility analysis” (p. 78). ^***c***^The IC2 framework allows for different levels of assessment; end points noted reflect all levels. The non-economic aspects of some external benefits are addressed in the IC2 Social Impact module.																					

Although several frameworks provide example tables of the cost considerations to be included in an alternatives assessment, details about data sources for the economic assessment are not included in the majority of the frameworks. Because most alternatives assessment frameworks have been developed to provide guidance to the business community, it may be presumed that cost-assessment methods are standardized, given the central need to perform such assessments as part of routine business practices. The Ontario Toxics Reduction Program’s framework provides a general overview of data source options for many of the outlined economic assessment end points (Ontario Toxics Use Reduction Program 2012).

Methods used for the comparative economic assessment of alternatives vary and are not always made explicit. The Lowell Center for Sustainable Production, IC2, and the Ontario Toxics Reduction Program reference the use of cost–benefit analyses (IC2 2013; Ontario Toxics Use Reduction Program 2012; Rossi et al. 2006). Four frameworks, including European Commission DGE (Gilbert et al. 2012), MA TURI (Eliason and Morose 2011; MA TURI 2006), UNEP’s POPs Committee (2009), and the TRGS 600 (BAuA AGS 2008) note options for using qualitative ranking methods when specific cost estimates may be missing, such as “better,” “neutral,” and “worse.” Others, such as the UCLA Sustainable Policy and Technology Program, report two summary measures: *a*) “manufacturer impact,” which estimates the extent to which expected revenues associated with the alternative are greater than manufacturing costs; and *b*) “purchaser impact,” which estimates the increased/decreased price paid by the consumer for the end product (Malloy et al. 2011, 2013). The UCLA framework’s use of “manufacturer impact” is similar in concept to “financial return on investment,” which is also noted as an option in the Ontario Toxics Use Reduction Program (2012).

The majority of alternatives assessment frameworks consider the alternatives as static options, with one notable exception being the IC2 framework. The IC2 framework includes a component in its cost assessment that allows the assessor to modify (possibly mitigating) negative cost and availability results through options such as purchasing contracts to achieve lower pricing, recycling of process chemicals to reduce quantities needed, or altering the product to incorporate alternatives in a more cost-effective manner (IC2 2013).

*Exposure characterization.* Eighteen frameworks include an evaluation of exposure (worker, public, and/or environmental) (Table 5). However, the manner in which exposure is addressed varies greatly. Seven frameworks include exposure characterization as a discrete process component—a specific step in the alternatives assessment process—whereas the remaining eleven typically address exposure to inform other process components, including focusing the hazard assessment, identifying priority uses of concern, informing the final selection of alternatives, and/or as a default decision-point if continued use of the chemical of concern is required because no safer and feasible alternative can be identified (Table 5). Nine frameworks consider exposure for purposes of characterizing risk. Some frameworks, such as BizNGO, do not consider exposure and associated risk assessment as an essential process component of alternatives assessment unless there are material, product, or process changes involved with adopting an alternative that could result in an exposure that is substantially different from the chemical of concern (Rossi et al. 2011). The NAS framework demonstrates an evolution in the consideration of exposure in alternatives assessment frameworks because it specifically includes a comparative evaluation of exposure to assess the potential for differential exposure as a result of differences between the chemical of concern and the alternative in terms of their physicochemical properties (e.g., differences in vapor pressure or persistence), exposure routes, and quantity used (NRC 2014). The NAS framework differentiates its methods from risk assessment, suggesting that the use of available exposure models or critical physicochemical properties is typically sufficient to determine the intrinsic exposure potential of alternatives relative to the chemical of concern (NRC 2014).

**Table 5:** Purpose of exposure characterization (*n* = 20).

Framework name (reference)	Exposure addressed?	Discrete process element?	Purpose	
			Risk characterization	Other (as described)
Goldschmidt 1993	✓		✓	Simply states, “assess the risk of being exposed.”
U.S. EPA CTSA (Kincaid et al. 1996)	✓	✓	✓
Rosenberg et al. 2001
Lowell Center for Sustainable Production (Rossi et al. 2006)				Inherent exposure properties and routes of exposure that substantively increase exposure levels are identified and integrated into the hazard assessment (human and ecological toxicity).
MA TURI (Eliason and Morose 2011; MA TURI 2006)	✓			Physicochemical properties are considered for worker exposure potential. Considered when identifying priority uses to include in the alternatives assessment and for comparing alternatives.
P2OSH (Quinn et al. 2006)	✓			Worker use conditions are characterized to identify exposure potential.
Royal Society of Chemistry (RSC 2007)	✓	✓	✓
TRGS 600 (BAuA AGS 2008)	✓		✓
UNEP Persistent Organic Pollutants Review Committee’s General Guidance on Alternatives (UNEP 2009)	✓	✓	✓
U.S. EPA DFE Program (Lavoie et al. 2010; U.S. EPA 2011a)	✓			Considered when applying life-cycle thinking to target exposure pathways of priority concern.
BizNGO (Rossi et al. 2011)	✓		✓	Use of risk assessment suggested only when alternatives differ from current practice. Addressed during the last step of the alternatives assessment process under Step 6, “Apply Lifecycle Thinking.”
German Guide on Sustainable Chemicals (Reihlen et al. 2011)	✓			Physicochemical properties considered for worker exposure potential. Releases/long-range transport considered regarding mobility and environmental exposure potential.
UCLA Sustainable Policy & Technology Program (Malloy et al. 2011, 2013)	✓			Characterized as part of subcriteria/end point within the hazard assessment (human health and environment). Considered the nature of exposure in comparison of alternatives, yet not for the explicit purpose of risk calculations.
REACH (ECHA 2011)	✓	✓	✓
U.S. EPA SNAP Program (U.S. EPA 2011b)	✓			Characterized exposure potential using physicochemical properties, use characteristics, emissions information and industrial hygiene information, yet not for the purpose of estimating risk.
European Commission DGE (Gilbert et al. 2012)	✓	✓	✓
Ontario Toxics Use Reduction Program 2012	✓			Considered primarily in the assessment of physicochemical properties and during the life-cycle assessment process.
OSHA 2013	✓			Worker use conditions are characterized to identify exposure potential. Characterized as part of subcriteria/end point within the hazard assessment.
Interstate Chemicals Clearinghouse (IC2 2013)^*a*^	✓	✓	✓	Exposure considered when examining potential trade-offs with the identified alternatives. In addition to risk assessment, several other options are offered that address exposure potential without estimating risk, such as physicochemical properties, use characteristics, emissions, and industrial hygiene information.
NAS (NRC 2014)	✓	✓		Included “intrinsic exposure” to determine whether exposure to the chemical of concern and alternatives are *a*) substantially equivalent; *b*) increased; or *c*) inherently (lower) preferable. More rigorous exposure assessment is suggested where increased exposure is indicated.
^***a***^The IC2 framework includes risk assessment only in the most rigorous exposure assessment process level (level 4).				

The vast majority of the frameworks evaluating exposure use indirect measures, such as dispersive potential or volume in commerce, rather than actual exposure models or data. Thirteen of the frameworks characterizing exposure link it to four particular categories of attributes: physicochemical properties, use characteristics, emissions and fate, and industrial hygiene measures (Table 6). Physicochemical properties are most often linked to exposure measures: vapor pressure/boiling point (*n* = 8), solubility (*n* = 6), physical state at room temperature (*n* = 6), density (*n* = 5), and dissociation constant (*n* = 3). As noted previously, physicochemical properties are also a core part of the hazard assessment process in the majority of the frameworks. Although some physicochemical properties, such as flammability or corrosivity, are clearly associated with the hazard profile of a substance (Table 2), others, including solubility, state (dust, gas, etc.), binding strength/migration potential, and vapor pressure, inform a substance’s inherent exposure potential (Table 6). Even environmental fate end points such as bioaccumulation (Table 6) are often predicted through physicochemical properties such as octanol–water partition coefficients. The NAS framework describes these and other physicochemical properties as intrinsic exposure properties (NRC 2014). Several frameworks, including those by the MA TURI, the Ontario Toxics Use Reduction Program, and BizNGO (using GreenScreen®), which do not include an explicit evaluation of exposure as a discrete step in the alternatives assessment process, do include several physicochemical properties that inform exposure potential in the hazard assessment process component (CPA 2014; Eliason and Morose 2011; MA TURI 2006; Ontario Toxics Use Reduction Program 2012).

**Table 6:** Exposure characterization attributes (most frequently addressed, not comprehensive) (*n* = 20).

Framework name (reference)	Physicochemical properties											Use characteristics				Emissions and environmental fate			Industrial hygiene	
	B–Binding strength/migration potentialD–Density/specific gravityDC–Disassociation constantDG–Dust-generating solids/aerosolsMP–Melting pointM/PS–Molecule/particle size MW–Molecular weightpH–pHPS–Physical state (at room temperature)S–SolubilityVP/BP–Vapor pressure/boiling point											A/C–Amount consumer useA/M–Amount manufacturer useD–Extent dispersive useP/H–Processing/handling characteristics				B/EM–Biomonitoring/environmental monitoringE–EmissionsPBT–Persistent, bioaccumulative, toxic			IH–Industrial hygiene controls OM–Occupational monitoring	
	B	D	DC	DG	MP	M/PS	MW	pH	PS	S	VP/BP	A/C	A/M	D	P/H	B/EM	E	PBT	IH	OM
Goldschmidt 1993
U.S. EPA CTSA (Kincaid et al. 1996)	✓											✓	✓		✓	✓	✓		✓	✓
Rosenberg et al. 2001^*a*^
Lowell Center for Sustainable Production (Rossi et al. 2006)^*a*^
MA TURI (Eliason and Morose 2011; MA TURI 2006)^*b*^	✓	✓								✓	✓
P2OSH (Quinn et al. 2006)															✓		✓
Royal Society of Chemistry (RSC 2007)																		✓
TRGS 600 (BAuA AGS 2008)				✓					✓		✓		✓		✓
UNEP Persistent Organic Pollutants Review Committee’s General Guidance on Alternatives (UNEP 2009)												✓	✓	✓	✓	✓	✓
U.S. EPA DFE Program (Lavoie et al. 2010; U.S. EPA 2011a)
BizNGO (Rossi et al. 2011)
German Guide on Sustainable Chemicals (Reihlen et al. 2011)	✓			✓		✓				✓	✓						✓^*d*^	✓
UCLA Sustainable Policy & Technology Program (Malloy et al. 2011, 2013)												✓	✓	✓				✓
REACH (ECHA 2011)^*c*^																	✓	✓
U.S. EPA SNAP Program (U.S. EPA 2011b)		✓	✓			✓	✓	✓	✓	✓	✓	✓	✓		✓		✓		✓	✓
European Commission DGE (Gilbert et al. 2012)		✓	✓	✓	✓		✓	✓	✓	✓	✓		✓		✓
Ontario Toxics Use Reduction Program 2012^*d*^											✓		✓				✓
OSHA 2013									✓						✓
Interstate Chemicals Clearinghouse (IC2 2013)^*e*^	✓	✓	✓	✓	✓	✓	✓	✓	✓	✓	✓		✓		✓	✓	✓	✓
NAS (NRC 2014)^*f*^		✓			✓	✓	✓		✓	✓	✓		✓					✓
These end points reflect those explicitly noted in the sources reviewed above beyond considerations such as routes and patterns of exposure. ^***a***^Exposure assessment not addressed. ^***b***^These measures are captured during the hazard assessment process. ^***c***^Specific exposure potential attributes not comprehensively outlined in the guidance materials, beyond referencing PBTs, “environmental fate properties,” and emissions. ^***d***^These measures are captured during the life-cycle assessment process. ^***e***^The IC2 framework allows for different levels of assessment; end points noted reflect all levels. ^***f***^The physicochemical properties are outlined in Step 5 of the NAS framework, which is a discrete step focused on such properties.																				

Use characteristics are outlined in 11 frameworks and capture information including processing and handling characteristics (*n* = 8) and manufacturer use amounts (*n* = 9) (Table 6). Frameworks concentrating on the workplace environment typically focus on use characteristics associated with occupational exposure (BAuA AGS 2008; OSHA 2013; Quinn et al. 2006). A few frameworks outline use characteristics that have broader public health and environmental implications for exposure, including amount in consumer use and extent of dispersive use (Table 6). Components associated with emissions and environmental fate (specifically PBTs) are included in 9 and 6 frameworks, respectively. Occupational monitoring data is one component that directly assesses worker exposure (rather than using surrogates of exposure) and is addressed in 2 frameworks (Table 6). The presence/need for industrial hygiene controls (e.g., ventilation, personal protective equipment) is also included in these frameworks (Table 6). Two frameworks, the Ontario Toxics Use Reduction Program (2012) and the German Federal Environment Agency’s Guide on Sustainable Chemicals (Reihlen et al. 2011), capture emissions/environmental releases as a part of the life-cycle component rather than as a part of exposure characterization.

The frameworks do not routinely recommend data sources for the exposure measures. When data sources are noted, SDSs and chemical encyclopedias are referenced for physicochemical properties, and public databases such as pollutant release and transfer registries and published literature are referenced for emission, fate, and transport information. The NAS framework refers to using publicly available exposure models to address identified exposure scenarios of concern (NRC 2014). Given the nature of the questions and guidance offered in the majority of the frameworks, expert judgment regarding work and environmental conditions that influence potential exposure appear to be a primary source of information. Exposure potential and/or risk are most routinely displayed as a qualitative (three-point or five-point) ranking rather than as quantitative statements of risk. For example, the European Commission DGE (Gilbert et al. 2012) framework uses information about where, how often, and in what way the chemical is used to rank exposure potential from 1 (low exposure) to 5 (very high exposure) with regards to working/process conditions, physical properties affecting exposure, frequency or duration of use, quantity used, and accident potential. Qualitative hazard and exposure potential scores are then combined to identify chemicals with the highest risk. The NAS framework describes an assessment of intrinsic exposure measures to determine whether likely exposure to the chemical of concern and alternatives is *a*) substantially equivalent, *b*) increased, or *c*) inherently (lower) preferable. Where the assessment of exposure indicates the potential for increased exposure, the NAS framework suggests that quantitative exposure assessment, although more complex and time-consuming than qualitative assessment, may be needed to discern between alternatives (NRC 2014). Hazard assessment tools, such as GreenScreen® (used in the BizNGO and IC2 frameworks), include the ability to stratify hazard severity scores by route of exposure in order to provide additional insight into factors that influence the ability of a chemical to cause harm (CPA 2014; Whittaker 2015).

Several frameworks, including those by IC2 (2013) and the European Commission DGE (Gilbert et al. 2012), outline questions for the assessor to consider mitigation options that could reduce exposure potential through, for example, process changes or upstream product design changes.

*Life-cycle assessment/life-cycle thinking.* Eighteen frameworks address life-cycle impacts (Table 7). There were two dominant approaches for addressing life-cycle impacts: life-cycle assessment and life-cycle thinking. Both follow the same general principle of thoroughly considering impacts at different points in the chemical/product life cycle to avoid selecting alternatives that shift risks from one stage of a product’s life cycle to another. Life-cycle assessment (LCA) follows a well-defined quantitative methodology, such as ISO 14040, that quantifies the impacts associated with a standardized set of environmental impacts (i.e., greenhouse gas emissions, resource depletion, water consumption, energy consumption) of products or processes across their life stages (ISO 2006). In contrast, life-cycle thinking is less analytical and generally less resource-intensive than LCA. Life-cycle thinking identifies significant impacts at different life-cycle stages but does not typically include quantitative assessment.

**Table 7:** Addressing chemical life-cycle impacts (*n* = 20).

Framework name (reference)	Life-cycle impacts addressed?	Addressed as a discrete process element?	General methods		
			Life-cycle thinking	Life-cycle assessment^*a*^	Other (as described)
Goldschmidt 1993
U.S. EPA CTSA (Kincaid et al. 1996)	✓		✓
Rosenberg et al. 2001	✓		✓
Lowell Center for Sustainable Production (Rossi et al. 2006)	✓		✓
MA TURI (Eliason and Morose 2011; MA TURI 2006)	✓		✓
P2OSH (Quinn et al. 2006)
Royal Society of Chemistry (RSC 2007)	✓
TRGS 600 (AGS 2008)	✓^*b*^	✓			References the use of “tried and tested expert method” for social, environmental, and economic end points.
UNEP Persistent Organic Pollutants Review Committee General Guidance on Alternatives (UNEP 2009)	✓		✓
U.S. EPA DFE Program (Lavoie et al. 2010; U.S. EPA 2011a)	✓		✓
BizNGO (Rossi et al. 2011)	✓	✓	✓^*c*^	✓^*c*^
German Guide on Sustainable Chemicals (Reihlen et al. 2011)	✓	✓	✓
UCLA Sustainable Policy & Technology Program (Malloy et al. 2011, 2013)	✓				Addresses 14 end points associated with life-cycle impacts.
REACH (ECHA 2011)	✓				References LCA for comparative evaluation of “far-reaching impacts,” yet states that LCA methods are not designed for the selection of lower-risk alternatives to hazardous chemicals associated with specific uses. Only alternative method offered is the Column Model.
U.S. EPA SNAP Program (U.S. EPA 2011b)	✓				Addresses environmental releases and exposure at specific life-cycle stages: manufacture, use, and disposal. Also interested in specific regulatory/programmatic end points, including ozone depletion and greenhouse gas emissions.
European Commission DGE (Gilbert et al. 2012)	✓		✓
Ontario Toxics Use Reduction Program 2012	✓			✓
OSHA 2013	✓		✓
Interstate Chemicals Clearinghouse (IC2 2013)	✓	✓	✓^*d*^	✓^*d*^
NAS (NRC 2014)	✓	✓	✓^*e*^	✓^*e*^
^***a***^Referencing accepted/standard life-cycle assessment methods. ^***b***^Not addressed in the typical assessment; part of an “extended assessment” for decisions that have far-reaching implications. ^***c***^Both methods mentioned, including their strengths and limitations. ^***d***^Life-cycle thinking is used in the preliminary and in levels 1 and 2; life-cycle assessment guided by ISO 14040 (ISO 2006) is referred to in level 2 and outlined as the main method in level 3. ^***e***^Use of life-cycle thinking is recommended before the use of life-cycle analysis to identify upstream and downstream impacts.					

The majority of the frameworks consider key life-cycle attributes in the context of hazard, exposure, economic, or technical feasibility assessments (*n* = 13) rather than as a discrete process component (*n* = 5). The IC2 framework and the NAS framework do both; life-cycle thinking is included as a discrete process component, and the results of the evaluation are intended to provide additional information to identify potential unintended consequences or to discern between alternatives (IC2 2013; NRC 2014). Four frameworks refer to using commonly available LCA methods and tools (Table 7). In all four frameworks, the use of LCA is considered to be an add-on process that may be the last step in evaluating candidate alternatives and that may help to differentiate the “safer” alternative or to identify potential unintended consequences of a substitution. However, several frameworks, including those that refer to using LCA, caution that conducting traditional LCAs can be very expensive and time-consuming. These frameworks also note that assessment is feasible for some end points such as energy consumption; however, data and analytic methods are lacking for others, such as occupational impacts in upstream manufacturing processes.

Although life-cycle thinking is reflected in the majority of the reviewed frameworks, some focus only on life-cycle considerations associated with the primary focus of the framework. For example, the OSHA (2013) and Rosenberg et al. (2001) frameworks, which focus on the work environment, consider occupational health and labor impacts across multiple life-cycle stages, yet they do not address broader environmental impacts, such as those commonly considered in LCA. The concept of “synthetic history”—the sequence of unit operations and chemical inputs that proceed from the acquisition of raw materials to the production of chemical intermediates to the production of the chemical of concern (or alternative)—is also elevated in the NAS framework as an important consideration to make explicit the impacts of building-block chemicals or byproducts that may not be present in the final chemical or product (NRC 2014).

*Decision making.* The decision-making approaches taken in the alternatives assessment frameworks can be analyzed across four dimensions: the decision function or purpose, the decision approach, the decision methods/tools, and the role of weighting. Decision function or purpose refers to the role that the alternatives assessment plays in the ultimate evaluation of the alternatives. As shown in Table 8, three frameworks have a comparative function, providing a structured way to compare the attributes of various alternatives against one another. Such frameworks identify trade-offs between the alternatives but do not offer guidance or direction for ranking the alternatives or for selecting a preferred alternative. Other frameworks provide a further selection/ranking function in order to identify a preferred alternative or set of alternatives or to rank the alternatives (*n* = 16). The remaining framework does not include a substantive discussion of decision making.

**Table 8:** Decision analysis (*n* = 20).

Framework name (reference)	Decision function			Decision approach					Decision tools/rules				Weighting	
	C–ComparativeSR–Selection/rankingN–None			Sq–SequentialSi–SimultaneousMx–Mixed (for screening—selection, type noted)Mnu–Menu					NarA–Narrative aloneS–StructuralA–Analytical					
	C	SR	N	Sq	Si	Mx	Mnu	NA/NS	NarA	S	A	NA/NS	Addressed	Method
Goldschmidt 1993		✓						NS				NS	NS
U.S. EPA CTSA (Kincaid et al. 1996)		✓				Sim–N/S			✓				NS
Rosenberg et al. 2001			✓					NA				NA	NA
Lowell Center for Sustainable Production, (Rossi et al. 2006)		✓						NS	✓				Implicit
MA TURI (Eliason and Morose 2011; MA TURI 2006)	✓			✓					✓				NS
P2OSH (Quinn et al. 2006)		✓				Sq–N/S						NS	Implicit
Royal Society of Chemistry (RSC 2007)		✓			✓				✓				Explicit/Qual	Elicited
TRGS 600 (BAuA AGS 2008)		✓			✓				✓				Explicit/Qual
UNEP Persistent Organic Pollutants Review Committee’s General Guidance on Alternatives (UNEP 2009)		✓						NS				NS	NS
U.S. EPA DFE Program (Lavoie et al. 2010; U.S. EPA 2011a)	✓							NA				NA	NS
BizNGO (Rossi et al. 2011)		✓				Sq–N/S				✓			Implicit
German Guide on Sustainable Chemicals (Reihlen et al. 2011)	✓							NA				NA	NA
UCLA Sustainable Policy & Technology Program (Malloy et al. 2011, 2013)		✓					✓				✓		Explicit/Quant	Elicited
REACH (ECHA 2011)		✓				Sq–Si			✓				Explicit/Qual
U.S. EPA SNAP Program (U.S. EPA 2011b)		✓			✓				✓				NS
European Commission DGE (Gilbert et al. 2012)		✓				Sq–Si			✓		✓		Implicit
Ontario Toxics Use Reduction Program 2012		✓				Sq–Si			✓		✓		Explicit/Quant	Elicited
OSHA 2013		✓						NS				NS	NS
Interstate Chemicals Clearinghouse (IC2 2013)		✓					✓			✓	✓		Explicit/Qual and Quant	Default/Calculated/Elicited
NAS (NRC 2014)		✓			✓					✓	✓		Explicit/Qual and Quant
Abbreviations: NA, not applicable because framework did not include a decision-making function; NS, nonspecified, meaning the framework did not discuss this dimension; Qual, qualitative; Quant, quantitative.														

The term “decision approach” refers to the general structure or order of the decision-making process for a particular point, such as screening (i.e., winnowing an initial set of potential alternatives) or generating a final ranking of alternatives. Existing alternatives assessment frameworks use three general decision approaches: sequential, simultaneous, and mixed (IC2 2013). The sequential framework considers one or more attributes, such as human health impacts, environmental impacts, economic feasibility, or technical feasibility, in succession. Any alternative that does not perform satisfactorily on the first attribute (which is often human health impacts or technical feasibility) is dropped from further consideration. The remaining alternatives are then evaluated with respect to the next relevant attribute, and the process is repeated until a preferred alternative or set of alternatives is identified. The simultaneous framework considers all or a set of attributes at once, allowing good performance on one attribute to offset less-favorable performance on another for a given alternative. The mixed framework is a combination of the sequential and simultaneous approaches. For example, if technical feasibility and economic impact are of particular importance to the decision maker, she/he may screen out certain alternatives on that basis using a sequential approach and subsequently apply a simultaneous framework to the remaining alternatives.

Seven of the frameworks in this review adopt no decision approach. Three of these frameworks do not substantively address decision making, and four address decision making generally but do not specify any particular decision approach. Six other frameworks adopt the mixed approach, using different approaches for screening potential alternatives and for generating a ranking of alternatives or preferred alternatives (See Table 8, column 5, under “Decision Approach”). For example, the Ontario Toxics Use Reduction Program (2012) uses a sequential approach for the initial screening of alternatives, and then applies a simultaneous approach to the remaining alternatives. Four other frameworks apply the simultaneous approach exclusively, including the NAS framework, which applies it first to screen alternatives based on human health impacts and ecotoxicity, and later for ranking alternatives based on a larger set of process components (NRC 2014). One framework applies only the sequential approach (Eliason and Morose 2011; MA TURI 2006). Finally, the IC2 and UCLA frameworks present the sequential, simultaneous, and hybrid approaches as a menu of choices without expressing a preference (IC2 2013; Malloy et al. 2011, 2013). The UCLA framework applies the various approaches in two case studies to illustrate how the choice of decision approach can affect the outcome of the alternatives assessment (Malloy et al. 2011, 2013).

**Figure 1:**
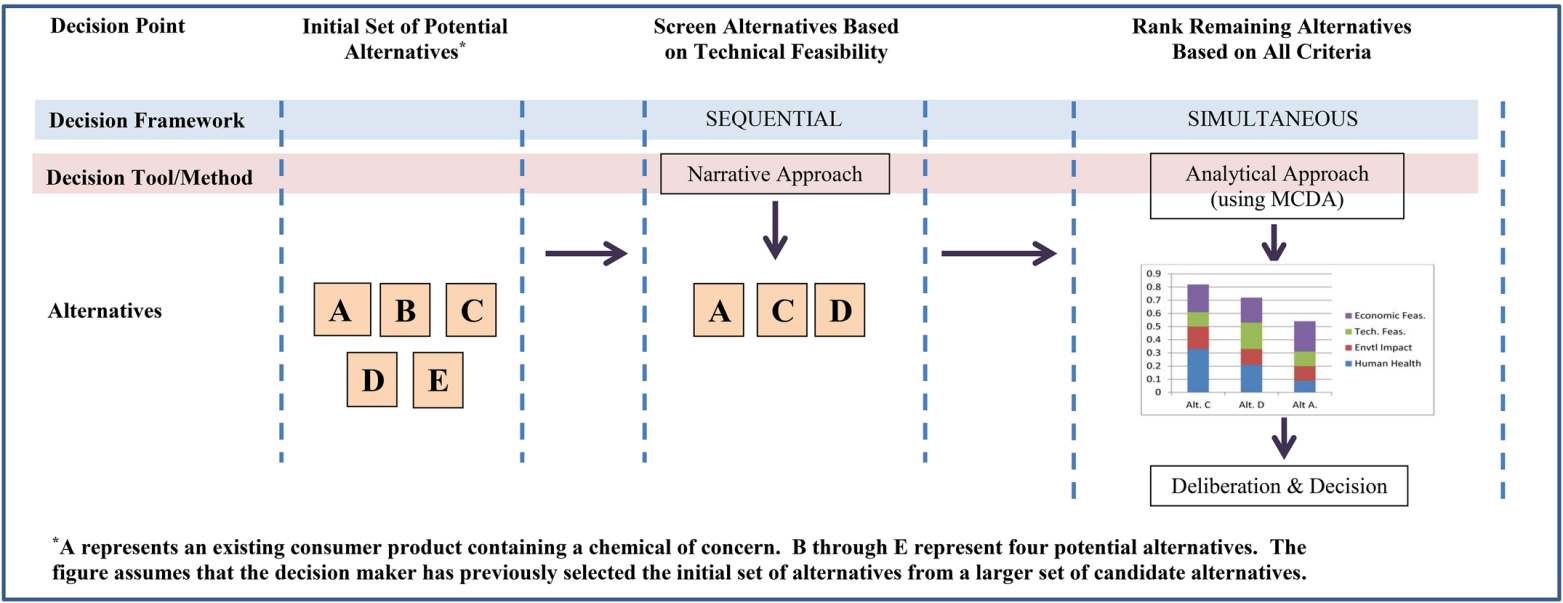
Example of a mixed approach: use of multiple decision tool in a mixed-decision framework (see Table 8 for details).

Decision tools or methods are formal and informal aids or rules that guide specific decisions, in this case the screening of alternatives and the selection or ranking of alternatives. Decision tools or methods can be separated into three general categories: narrative, structured, and analytical. With narrative methods, the decision maker engages in a holistic, qualitative balancing of the data and associated trade-offs to arrive at a selection. In some cases, the decision maker may rely upon explicitly stated informal decision principles or expert judgment to guide the process. Structured approaches apply a systematic overlay to the narrative approach, providing the analyst with specific guidance about how to make a decision. The structure may take the form of a decision tree, which takes the analyst through an ordered series of questions. Alternatively, it may offer a set of specific decision rules or heuristics to assist the analyst in framing the issues and guiding the evaluation. Analytical methods similarly function as a supplement to narrative approaches, using mathematically based formal decision analysis tools such as multicriteria decision analysis (MCDA) (Linkov and Moberg 2011). MCDA consists of a range of different methods and tools, reflecting various theoretical bases and methodological perspectives. Accordingly, these tools tend to assess data and generate rankings in different ways (Kiker et al. 2005). Figure 1 illustrates a mixed decision approach using two decision methods in sequence: a narrative method followed by an analytical method.

Nine of the frameworks rely upon narrative methods alone. Some of those nine frameworks provide general principles to guide the decision making. For example, the Lowell Center framework includes general principles (i.e., consider prevention, precaution, substitution, and a life-cycle perspective) and preferences (e.g., prefer solutions that eliminate the function of problematic chemicals). Other narrative frameworks offer little in the way of guidance for the decision maker. Still other frameworks, such as the BizNGO framework (Rossi et al. 2011) and the IC2 framework (2013), go beyond narrative alone to provide well-defined, structured decision approaches. The NAS framework also encourages the use of structured approaches in appropriate circumstances. Five frameworks, including the NAS, IC2, and UCLA frameworks, incorporate analytical methods as support tools for decision makers (Table 8). Four of the five frameworks using analytical tools focus on MCDA tools, whereas the European Commission DGE (Gilbert et al. 2012) framework relies upon cost–benefit analysis. The seven remaining frameworks either do not include a decision-making function or do not specify particular tools or methods.

The last dimension of interest is the extent to which the various frameworks engage in weighting of the decision criteria. In most situations, decision makers are not equally concerned about all decision criteria. For example, a decision maker may place more importance on whether a household cleaner causes cancer than on whether it contributes to smog formation. The reviewed decision frameworks handle questions of whether and how to weight criteria differently. Nine of the frameworks do not address the question of weighting at all. Three of the frameworks (Table 8) establish implicit weighting through the use of sequential decision approaches: by situating a criterion early in the decision sequence, the framework gives it greater influence on the ultimate decision. The decision structure created by the BizNGO framework also implicitly gives a specific set of chemical hazard end points greater weight (Rossi et al. 2011). Seven other frameworks call for explicit consideration of the relative importance of the decision criteria; four of those frameworks encourage development of quantitative weights where appropriate (Table 8).

## Discussion

In response to regulatory, business, and consumer drivers to substitute chemicals of concern in a wide array of products and processes, governments, NGOs, and academic researchers have developed alternatives assessment frameworks to aid in identifying, evaluating, and implementing safer substitutes (Edwards et al. 2011). This substantive review indicates that alternatives assessment is a growing field of science policy assessment, with established frameworks and an increasing number of tools and resources to support its practical application. Indeed, the growth of alternatives assessment frameworks demonstrates an increased recognition of the importance of an informed transition to safer alternatives as a key aspect of chemicals management science and policy. The alternatives assessment frameworks analyzed in this review share a common purpose: namely, identifying safer alternatives based on comparative assessments of hazard (and sometimes exposure) characteristics as well as technical and economic feasibility. This purpose—supporting a transition to safer alternatives while avoiding unintended consequences of uninformed substitutions—underscores the action or solutions orientation of alternatives assessment processes. The NAS framework specifically distinguishes alternatives assessment from other processes such as risk assessment, safety assessment, and sustainability assessment (NRC 2014).

This review identified 20 alternatives assessment frameworks that have been published since 1990. The NAS framework and a recent report by OECD reviewed 10 and 8 frameworks, respectively (NRC 2014; OECD 2013). The only framework not included in our review that was noted in the NAS report was the framework established under the California Safer Consumer Products program, for which, as of this writing, the California Department of Toxic Substances Control has not published its guidance framework other than requirements outlined in the regulation (CA Code of Reg 2013). Thus, we are confident that our search strategy retrieved a broad collection of relevant frameworks for evaluation. The additional frameworks identified by this search include historical frameworks (Goldschmidt 1993; Kincaid et al. 1996); frameworks used in additional regulatory programs, such as U.S. EPA’s Significant New Alternatives Policy (SNAP) program associated with alternatives to ozone-depleting chemicals (U.S. EPA 2011b); and frameworks used in occupational safety and health research and programs (BAuA AGS 2008; OSHA 2013; Quinn et al. 2006; Rosenberg et al. 2001). This review strictly required alternatives assessment frameworks to include at minimum an assessment of hazards, costs, and performance, which is consistent with the NAS framework and the OECD report (NRC 2014; OECD 2013). Our findings are relevant only to the alternatives assessment frameworks so defined. Although the alternatives assessment field may incorporate an array of science policy fields and disciplines—for example, life-cycle assessment and risk assessment—the findings in this review are not intended to be generalizable to these fields. However, the review does speak to how aspects of these fields have been adapted for use in the context of chemical alternatives assessment.

Our review identifies an important need for enhanced consistency in terms of particular methods, end points addressed, and evaluation criteria (i.e., ranking and scoring criteria). That said, the flexibility to adapt a transparent alternatives assessment process to different decision contexts is also needed, including articulating the circumstances under which particular methods and approaches are most appropriate. Although the hazard assessment component demonstrated the greatest area of methodological consistency among the frameworks reviewed, achieving increased consistency within a core set of hazard, economic, and technical feasibility characteristics as a baseline for any alternatives assessment should be explored. The IC2, European Commission DGE, and TRGS 600 frameworks offer useful models for providing a “core” or “minimal” set of attributes for the various process components that respond to the business community’s needs to conduct alternatives assessments that are more streamlined and that minimize time and resource requirements—a challenge for small and medium-sized companies (BAuA AGS 2008; Gilbert et al. 2012; IC2 2013).

An important research need is an evaluation of the outcomes of various alternatives assessment frameworks to understand the degree to which different frameworks and a minimum core set of end points (included in various forms in the six alternatives assessment process components) lead to significant differences in the identification of safer, feasible alternatives. Such an evaluation could identify core end points and data required to ensure a thorough evaluation of alternatives that minimizes the potential for unintended consequences, given that no framework or assessment can provide certainty about the impact of trade-offs. Indeed, the risk assessment literature clearly demonstrates that no assessment method can provide perfect consistency in outcomes because assessment results can differ greatly based on disciplinary perspective and data sources (Bailar and Bailar 1999). As noted in the NAS framework, a set of steps that ensure broad thinking about the potential consequences of a substitution, combined with transparency in methods and decision rules, are critical elements of any alternatives assessment (NRC 2014). Although this review focused on frameworks for alternatives assessment, there is a growing body of alternatives assessments that have been conducted using some of these 20 frameworks (IC2 2013). For example, numerous alternatives assessments have been conducted by industry using ECHA’s framework in order to comply with chemical authorizations requirements under REACH in the EU (Vainio 2015). Evaluation of such alternatives assessments is needed to gauge the real-world implementation of such frameworks. Research on existing and newly developed alternatives assessment case studies would allow for carefully structured investigation of specific methodological issues and potential solutions.

Methods are more developed in the hazard assessment component than in other components, yet gaps remain. For example, additional methodological development is needed to incorporate a broader array of ecotoxicity end points than is currently included (NRC 2014). Aquatic toxicity was generally the only ecotoxicity end point included, if at all, in the frameworks evaluated herein. An additional significant barrier affecting the assessment of chemical hazard is the lack of hazard data (Whittaker and Heine 2013). Many alternatives assessment frameworks rely on SDSs or GHS hazard phrases. These sources may lack important data relevant for specific hazard end points. Moreover, given that the U.S. NTP has only conducted 2-year carcinogenicity bioassays on approximately 600 of the tens of thousands of chemicals that are presently being used in commerce, data gaps for critical end points, such as carcinogenicity, are a significant issue confronting informed chemical substitution (NTP 2014b).

Several alternatives assessment frameworks identify a number of strategies to address data gaps, including use of heuristics and qualitative and quantitative structure–activity relationship models, in order to avoid substitutions where information about health and safety is missing (BAuA AGS 2008; CPA 2014; ECHA 2011; Lavoie et al. 2010; U.S. EPA 2011a). There is a need to augment data sources available for alternatives assessment (Lavoie et al. 2010). Such enhancement includes harnessing the potential in emerging forms of predictive toxicology, including high-throughput *in vitro* assays and advanced chemical informatics tools to combine data from multiple sources (NRC 2014). This enhancement could also include the use of probabilistic models and decision analytical tools for managing uncertain data (Malloy et al. 2013). Ultimately, given market and regulatory pressures, substitutions will be made, and it is important that data are available to inform efficient alternatives assessment processes.

Reform of federal chemicals policies to require chemical manufacturers to provide data on the hazards of the chemicals they are bringing to market, and to chemical users on their various uses, as required under the EU REACH regulation, could go a long way to address these data gaps. In the United States, reform proposals currently under consideration in the House and Senate provide the U.S. EPA with the authority to require needed testing when reviewing new and existing chemicals (Frank R. Lautenberg Chemical Safety for the 21st Century Act 2015, https://www.congress.gov/bill/114th-congress/senate-bill/697; Alan Reinstein and Trevor Schaefer Toxic Chemical Protection Act 2015, https://www.congress.gov/bill/114th-congress/senate-bill/725/).

Additional methodological and data gaps are notable in the exposure characterization, life-cycle assessment, and decision-analysis or decision-making process components. To date, exposure assessment has been primarily employed in risk assessment. This use of exposure assessment may remain a requirement, particularly for regulatory alternatives assessment frameworks in which risk estimates must be calculated or when companies adopting alternatives also need to demonstrate “safety” for a regulatory agency. There is a need to create methods for characterizing exposure that can inform substitution processes, including evaluating the hazard profile of a given alternative, identifying potential unintended consequences of substitutions, and improving our understanding of what is “safer.” The NAS framework considers the role of exposure in the alternatives assessment process and offers a starting point for future research on substitution-oriented exposure characterization (NRC 2014). The majority of frameworks, including those that are “risk-based” and “hazard-first,” include exposure metrics, primarily physicochemical characteristics and use/handling characteristics. Thus, current frameworks include methods that consider the intrinsic exposure properties of a given chemical or material and therefore inform the inherent hazard profile. Exposure data at the population level, however, are sparse and most likely would not be helpful in the evaluation of chemical substitutes. Methods to rapidly characterize and categorize potential exposures are needed. For example, the development of “E” (exposure) phrases that that identify intrinsic exposure, similar to the “H” and “R” phrases used by GHS, would be advantageous to the exposure evaluation process in alternatives assessment.

With regards to evaluation of life-cycle impacts, the most developed methods are in frameworks that employ LCA. However, the existing LCA methodologies have limitations in the selection of safer alternatives: most notably, the resource intensiveness of a standard LCA approach, the lack of toxicity data on many chemicals, and a lack of data on the release of chemicals during the product-use phase. Thus, the majority of the reviewed frameworks use a less well-defined, life-cycle thinking approach. What is clear in the rationale for adopting life-cycle thinking is the need for a more streamlined approach to identifying life-cycle impacts. However, greater methodological clarity about what is encompassed in life-cycle thinking would be of benefit to the alternatives assessment field. A body of literature that explores the use of comparative life-cycle assessment for the purpose of identifying alternatives is now available (Zhou and Schoenung 2007; Kikuchi et al. 2011). A deeper examination of how these methods could be more broadly incorporated and standardized in current alternatives assessment frameworks should be performed.

Our review identified two key findings regarding the decision-making component of an alternatives assessment. First, formalized decision-making processes in alternatives assessment require significant development; almost half of the reviewed frameworks do not consider the ultimate evaluation of trade-offs and the selection of preferred alternatives. Many of the frameworks that consider decision making provide little in the way of guidance. Second, there is a rich variety of approaches available to support decision making for alternatives assessment, and some of these approaches have been put to use in existing alternatives assessment frameworks. Identifying the “best” decision-making approach in a given setting is itself a thorny decision that will require further research in three areas. From the empirical perspective, it is important to gain a full understanding of the impacts that various decision approaches have upon alternatives assessment outcomes. For example, how do sequential versus simultaneous frameworks affect decision outcomes? In addition, from the normative standpoint, it would be helpful to develop design principles for alternatives assessment and to explore how different approaches, decision frameworks, methods and tools, and weighting may affect those principles and under what circumstances. Finally, from a methodological perspective, we should develop approaches for “validating” alternatives assessment methodologies against normative principles. This process will involve “operationalizing” our normative principles to engage in rigorous evaluation of our alternatives assessment frameworks.

With regards to the economic and technical feasibility components of an alternatives assessment, our analysis identified a number of different ways in which these elements are addressed in the various frameworks. This variety has two likely explanations. First, regulatory requirements, such as those in Europe and California, may dictate the types of economic considerations that must be included in an alternatives assessment; second, technical feasibility and cost assessment tend to be context- and firm-dependent (CA Code of Reg 2013; European Parliament and Council 2007). Performance requirements are often identified by purchasers or manufacturers and are assessed differently by different firms and sectors. Furthermore, different firms may have different return on investment requirements or manufacturing costs that make single economic assessment approaches a challenge. Frameworks such as the IC2 and TRGS600 frameworks outline generic cost and performance considerations/questions that can be included in alternatives assessment processes (BAuA AGS 2008; IC2 2013).

Although additional research and methodological development are needed to advance the practice of alternatives assessment, it is important that the processes continue to be flexible and adaptable to different contexts. An assessment process that is overly resource-intensive, costly, or slow will likely not be widely adopted, which would undermine the goal of alternatives assessment in supporting an informed transition to safer, feasible alternatives. Broadening alternatives assessment processes to include process components such as life-cycle impact evaluation and exposure is important for expanding the horizons of thinking about potentially costly and unintended consequences of substitutions. It is equally important that research be performed to identify assessment tools and approaches that can be readily used by a wide range of actors to facilitate efficient alternatives assessment processes.

## Conclusions

Alternatives assessment did not arise fully formed as a new methodology or approach to assessing substitutions for chemicals, materials, or activities of concern. The roots of alternatives assessment are found in decades of environmental impact assessment, technology assessment, and pollution prevention planning. However, the field has evolved quickly in recent years because of increasing scientific, policy, and market attention to chemicals of concern used in manufacturing processes and everyday products. As a result, a number of new frameworks and tools have been created to address this growing need. The growth in different approaches, which respond to varied drivers and contexts, is an understandable and logical consequence of increased attention to chemical substitution.

Significant similarities and some important differences in how the various alternatives assessment components are addressed were revealed for the twenty frameworks examined in this substantive review. We conclude that there is a need for increased consistency between frameworks, particularly in how hazard end points are evaluated and how exposure is addressed, while maintaining sufficient flexibility to allow the alternatives assessment process to be adapted to different decision contexts and resource availability. Ultimately, although there may be differences of opinion about what constitutes an adequate alternatives assessment, what is of key importance is that the assessor at least considers and evaluates, to the highest degree possible, the various process components. Because the goal of alternatives assessment is to support an informed transition to safer chemicals, materials, and products, breadth of consideration may in some cases be more important than the depth to which any particular process component is evaluated. Indeed, excessive depth of analysis in any one of the process components may lead to inaction and could undermine the solutions-oriented objective of alternatives assessment.

Our review also identified specific research needs. However, methodological research and development must consider the varied contexts in which alternatives assessment will be used. Many alternatives assessment practitioners, particularly those in smaller firms, do not have significant technical or financial resources to conduct detailed quantitative assessments (e.g., of exposure or life-cycle impacts). There is a need for approaches that are thoughtful, yet time- and resource-efficient, as well as for technical and research support for those conducting assessments. There is also a critical need for enhanced hazard, exposure, and life-cycle data in “actionable” formats to complete alternatives assessments.

Some may argue that alternatives assessment should not be practiced on a large scale until issues of consistency and research gaps are addressed. The evolution of alternatives assessment, however, is no different than the evolution of other science policy approaches, such as that of risk assessment. The publication of the National Research Council “Red Book” in 1983 stimulated years of discussion that led to the growth of the risk assessment field and to additional NAS studies, guidance, and efforts at standardization (NRC 1983). During this period, risk assessments were conducted and improved, and the field grew. Given that decisions regarding chemical substitution are being made by governments and companies in the present day, the coming years will see a need for greater collaboration on methods development and standardization of approaches that can maintain the core goals of alternatives assessment to support efficient, informed decision making. This process will by necessity be iterative.

We conclude that alternatives assessment is a growing field of scientific assessment with rigorous methods and tools. The multi-disciplinary nature of alternatives assessment requires enhanced scientific collaboration across fields to refine methodologies that can support the important sustainability goal of informed substitution and design of safer chemicals, materials, and products.
